# Perforation of the Intestine in Typhoid Fever

**Published:** 1903-05-30

**Authors:** 


### "PERFORATION OF THE INTESTINE IN TYPHOID FEVER."

PERITONITIS, the result of a perforating ulcer of the intestine, occurs in about 3 per cent. of cases of typhoid fever.[1] The perforation is nearly always in the lower part of the small intestine, and usually it is within two feet of the ileo-cæcal valve. Sometimes, though rarely, the colon is the seat of perforation, and occasionally the vermiform appendix. The complication occurs more often in adults than in children, and more frequently in men than in women. It may take place in the mildest cases as well as in the most severe, and may occur in the first attack or during a relapse, or when the patient is convalescent, but it seldom happens before the third week of the illness. In walking typhoids the sudden onset of severe abdominal pain and other accompaniments of perforation, may be the first symptoms for which the patient seeks medical advice. As regards causes, great distension of the bowel with gaseous products of decomposition, and the presence of undigested food, are perhaps the most important. Movements on the part of the patient, and obstinate vomiting, may also lead to perforation; but it sometimes occurs in the absence of any of these conditions. It is not infrequently associated with hæmorrhage. With regard to the post-mortem appearances the intestine may be extensively ulcerated, or the ulcers may be very few. The parts near the ileo-cæcal valve are more markedly affected than those higher up. Sometimes there are as many as three perforations, but usually there is but one, which may be as minute as a pin's head, or larger than a sixpenny-piece. In some cases the edges of the perforation may be found imperfectly sealed by a tag of great omentum. In other cases there are no adhesions to the perforation. The peritoneum around the perforation shows signs of acute inflammation, which may be localised, or more often, is a part of a general septic peritonitis. Though the symptoms of perforation are somewhat variable in degree, and in order of occurrence, their onset is always sudden. From the time that perforation takes place the patient's general condition becomes progressively worse. The first symptom usually is sudden severe pain in the abdomen, most frequently in the lower part. The pain is persistent, and rapidly becomes general over the whole of the abdomen, though in some cases it may be more like colic. Rarely pain is entirely absent. Vomiting usually sets in early, during the first hour or two, but may not come on until the other signs of peritonitis are well marked. An invariable sign of perforation is an increase in the pulse rate to over 120 per minute. With the pulse the respirations are also increased in frequency. The temperature is not nearly such a valuable guide, but some sudden fluctuation is almost always to be found. There may be a sharp fall, followed by a rapid rise, or there may be the rapid rise without any initial fall.[2] The abdomen, if retracted at first, becomes distended; if already distended when perforation occurs, the distension increases, thus embarrassing respiration. The movements of the abdomen during respiration become impaired, and finally cease, so that the breathing is wholly thoracic, while the abdominal walls become quite immobile. On percussion the note in front is tympanitic. The absence of liver dulness, if the abdomen is retracted, is strong evidence of free gas in the peritoneum, but it is far from being a reliable sign. Dulness in the flanks occurs late. The abdomen is tender to touch, sometimes very slightly, sometimes excessively. The tenderness is general; though it may commence in one iliac fossa, it does not remain localised there. Rigidity of the abdominal muscles, especially if it is constant, and on one side only, is an important early sign, and may be found before tenderness begins. On auscultation, friction rales may be detected over the peritoneum. Examination per rectum, reveals increased heat and tenderness most marked in the region of the recto-vesical pouch of the peritoneum. As the patient's condition gets worse, the prostration increases. His face becomes pinched, and covered with beads of sweat, the corners of the mouth drawn, the eyes sunken and dull, and the skin feels cold and clammy. The pulse becomes irregular and is felt only with difficulty. He gradually sinks, the mental condition often remaining clear to the end.

If the symptoms be due to merely a leaking typhoidal ulcer, they may not be very characteristic. The pulse, however, will rise and the temperature may fall, and the respirations become thoracic. There may be vomiting and a little increasing pain. In the most malignant type of typhoid, the symptoms of perforation, may be entirely masked. The difficulties which surround the diagnosis of perforation and peritonitis may be very considerable. Fortunately in some cases the diagnosis is more easily made. In a walking typhoid the sudden onset of abdominal pain, vomiting, accelerated pulse and respirations, tender rigid abdomen, may be mistaken for, *e.g.*, a case of acute appendicitis. A history of slight malaise, lasting over a fortnight, or of contact with some source of infection, may, however, be obtainable. In mild cases of typhoid, when the patient is already under treatment, diagnosis is easier, owing to the opportunity of noting any sudden and rapid change in his condition. On the other hand, if during the course of the fever there has been troublesome vomiting, much colic or distension, perforative peritonitis may be unrecognised until too late. In such cases the pulse is the most valuable guide. Generally speaking, hæmorrhage and perforation are the two causes for rapid acceleration in pulse rate. If there be no external bleeding, and no pallor, hæmorrhage can be dismissed, and the greatly accelerated pulse rate ascribed to perforation. In malignant typhoid with low muttering delirium, the rapid increase in pulse and respiration rate may be thought to be due to the onset of pneumonia, when in reality, as an autopsy would reveal, it is due to perforation. If the pulse rate remains unaltered, an attack of very severe pain in the abdomen, even if so severe as to cause profuse sweating and collapse, is probably due to colic and not to perforation. The greater number of cases of perforation end fatally. Spontaneous recovery is very rare. Abdominal section is the most successful form of treatment.[3] A sufficient number of successful cases has been recorded to direct special attention to this measure.[4] If the operation is to be successful, it must be done at the earliest opportunity.[5] The longer the delay the less the patient's chance of recovery. The administration of opium for the purpose of diminishing peristaltic movements and limiting intestinal extravasation has been long practised, but its good influence is lessened by the distension of the intestines it produces, and indeed success by drugs alone is rarely achieved. By laparotomy the perforation is closed, and the extravasated intestinal contents are removed and the peritoneum drained. The distension of the intestines can be relieved by puncture if deemed necessary during operation. The earlier the perforation is recognised and the operation performed the greater the chance of success.

[1] Allbutt's System of Medicine, Vol. I. p. 823. [2] A Civilian War Hospital (Murray, London), p. 89 et. seq. [3] Hare, Complications of Typhoid Fever (1899). [4] Brit. Med. Jour., Heuston, Vol. II., p. 1454 (1901). [5] Ibid., Clin. Soc., Bowlby and Waring, Vol. II., p. 1900 (1902).

